# Mathematical model for footwear upper material consumption estimation

**DOI:** 10.1016/j.heliyon.2024.e31046

**Published:** 2024-05-15

**Authors:** Muhammad Naimul Hasan, Md Elias Uddin, Anower Jahan Tamanna

**Affiliations:** Department of Leather Engineering, Faculty of Mechanical Engineering, Khulna University of Engineering & Technology, Khulna, 9203, Bangladesh

**Keywords:** Footwear industry, Upper material consumption, 2D nesting norm, Waste minimization, Sustainability

## Abstract

Appropriate material consumption estimation since the design phase for footwear fabrication is a vital issue as material costs account for a sizeable portion of the overall production cost of a pair of shoes. This paper presents a mathematical model to predict the calculation of footwear upper material consumption to improve the utilization ratio of materials through a suitable nesting map onto leather. The proposed model concentrates on the two-dimensional geometry of footwear components and the application of rich mathematical concepts. The model reflects the outlines of individual footwear components while determining the area using definite integral calculus. Five distinct rotational kinds are applied for component arrangements that correlate to the physiognomy of leather because nesting onto the leather is intractable. The simple concept of a minimal-area polygonal enclosure is applied to maximize material utilization with minimum waste. Finally, the model was verified for four consecutive Oxford footwear sizes by comparing actual upper material consumption with predicted upper material consumption. The noble contribution of this analysis is to use ImageJ software to compute upper material consumption in real case analysis through image processing techniques and separate estimation of wastes, especially the fourth waste. The results of the comparison study show that the proposed model can reduce average material requirements by 2.06 %. This minimization of waste could be beneficial in terms of economic and environmental sustainability. Therefore, this study can be applied to estimate more accurately the amount of upper material required for footwear fabrication and support better utilization of material in the footwear industry**.**

## Highlights

• A mathematical model for footwear upper materials consumption computation is proposed for the footwear industry.
• Anticipated certain optimized nesting of footwear components onto leather to minimize waste.
• The average material utilization achieved by this proposed model is 73.89 %.
• The proposed model could minimize average material requirements by 2.06 %.

## Graphical abstract

**Figure:**
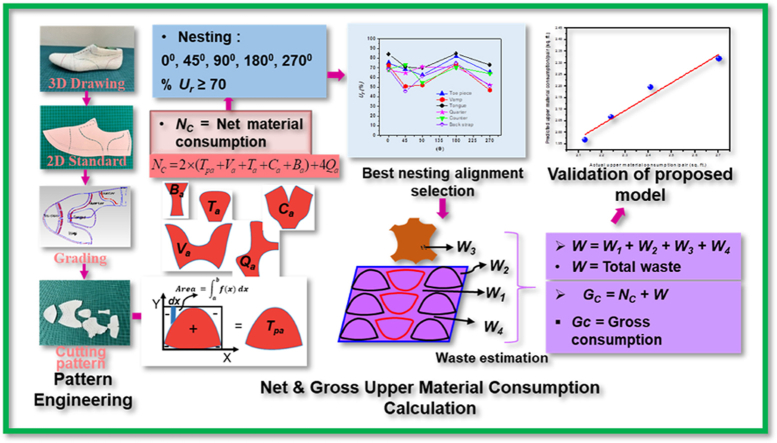

## 1 Introduction

Estimation of footwear material consumption is principally focused on the quantity of material required to fabricate a pair of foot apparel with an attitude to maximize raw material utilization in an economized and sustainable way in the footwear industry. Since the design phase, cutting materials into the required irregular shapes of smaller pieces is a fundamental phase of many manufacturing processes, and the footwear industry has been reflected as a prominent one facing nesting difficulties with cutting wastes [[1], [2], [3]]. Annually 4.46 × 10^5^ tons of waste from leather upper and lining materials and 2.01 × 10^3^ tons of waste from non-leather upper and lining materials are generated from the cutting unit of the footwear industry [4]. Jadhav, N.C., Jadhav, A.C. (2020) [5] stated that every year 134 tons of waste per million pairs of footwear upper material is generated from the cutting section of footwear industries. Suitable material consumption appraisal through perfect placement of components onto the material surface for any product is highly desirable to curtail material waste as the cost of material is an integral portion of manufacture cost [6,7].

The components of foot apparel are comprehensively organized into three parts: the upper, bottom, and the grindery. The upper parts over the sole consist of the toe piece, vamp, quarter, counter, back strap, etc., and the bottom parts comprise the insole, midsole, and sole [8,9]. The cutting of upper components from material is one of the vital stages of the shoemaking process [10]. However, around 20–45 % of waste is generated in a cutting unit of the footwear industry because of material grades, inefficient nesting, and cutting practices [4,5]. The footwear fabrication consumes more than 65 % of the world's total leather production [5,11] and the upper parts incur 70 % value to the overall price of a pair of footwear [12]. Global footwear production has stretched around 23.9 billion pairs in 2022 [13] with a market value of US$ 377.7 billion. It is anticipated to stretch a size of US$ 440.1 billion by 2030 [14]. Bangladesh shares over 1.6 % of the world's total footwear consumption [13]. The leather and leather products sector added more than US$1.2 billion of export revenue yearly for Bangladesh [15]. Consequently, the emergence and the economic expansion of the footwear industry have driven numerous Bangladeshi manufacturers to expand their production facilities to produce a diverse range of footwear with versatile upper materials.

According to studies in literature, roughly 40 distinct materials have been identified in the cutting room of the footwear industry, of which Around 25 % of the upper materials are still leather [4,5]. Leather as a natural material differs not only in its mechanical properties but also in its physiognomy [16]. Nesting footwear components onto the leather is intractable as the footwear components and leather both are unusual in contours [17]. Besides, the quality of the leather is not even all over the surface; certain regions are only appropriate for specific types of footwear components [10]. Additionally, leather hide bears tightness and stretchiness directions which hinder some footwear components from being nested in at definite angles [17,18]. Therefore, upper material consumption estimation pertaining to the perfect nesting of footwear parts considering the leather physiognomy has become the hardest [19] and most challenging for the footwear manufacturer.

For maximizing material utilization, diverse methods, for instance, No Fit Polygon (NFP) [2], Particle Swarm Optimization algorithm (PSO) [3], Guided Cuckoo Search (GCS)**,** pairwise clustering [7], and genetic algorithm [20] as heuristic tools for parts sequencing have been explored in the glass, paper, garments, and many other industries [2,3,7,20,21]. NFP is a geometry algorithm effectively used for solving the 2D packing difficulty of numerous shapes. NFP can work efficiently only when the convex shapes are considered otherwise it is difficult with arrangement limitations [2,21]. PSO algorithm is widely used in the garments industry but it deals with the large memory requirements and high time to search desired results for patterns nesting [3]. Suitable nesting is to some extent, difficult through GCS as there is a possibility of erroneous selection because the nest is chosen on a random basis and the quantity of available host nests is fixed [7]. Nevertheless, the cutting of shoe components is barely automatized [10]. Subsequently, nesting and cutting of footwear parts manually from leather is centuries of practice and it is due to natural zoning and direction of stretchiness [17].

A group of researchers focused on geometrical methods to estimate footwear materials consumption. Russ and Small's Method (RSM), Scientific Leather Measurement (SLM) [22,23], and the marking up method [24] are the most widely used consumption estimation techniques in footwear fabrication. Furthermore, shoemakers employ traditional CAD-CAM software based on the RSM principle [25,26]. Only the 0^0^ and 180^0^ sets of nesting arrangements are thought to exist in the aforementioned ways, and not even estimating the first waste individually in consumption calculation, they usually ignore the fourth waste (the gap between cutting dies). The software systems are also rigid, expensive, and require a lot of upkeep. Therefore, inferring better nesting difficulties augmentation to some extent and possibility of over or under consumption. On the other hand, industrial engineering has tried to grasp the behavior of production structures to control waste at the source, though entire waste eradication is not realistic. Recently, concentration has been developed in applying tools from mathematical science to the design, progress, and engagement of engineering procedures to optimize industrial methods. In this research, a mathematical model is proposed to predict footwear upper materials consumption by addressing the complex nesting problem in the cutting section of the footwear industry. The main purpose of this research is to minimize the cutting waste for leather consumption per pair of shoes by improving nesting efficiency. The research focuses on exploring the performance of nesting efficiency with multiple orientations for two-dimensional (2D) irregular footwear components regarding leather surface physiognomy to reduce leather consumption in the footwear fabrication process.

The succeeding structure of the paper is as follows: Section 2 describes in detail the problem description of footwear components with nesting difficulties, model formulation, and the search for perfect nesting alignment through the setup rule of nesting variants for maximum utilization ratio. Then estimation of associated wastes from the best nesting map is carried out to compute gross consumption. This section ultimately describes the sequential stages of the model formulation to compute material consumption. Section 3 deals with the computational outcomes and discussion, real case analysis through image processing technology for model validation, and a comparison study to evaluate the magnitude of contribution for this analysis. Section 4 draws concluding remarks along with future research directions.

## 2 Problem statement and model formulation

This part addresses the problem related to irregular pattern nesting of footwear fabrication, and the model formulation process to compute gross consumption. This section introduced how to get an optimal nesting alignment of complicated geometries of footwear components to minimize component interlocking waste. Therefore, a complete guide to computing upper material consumption is represented in this paper.

### 2.1 Problem statement

**Fig. 1:**
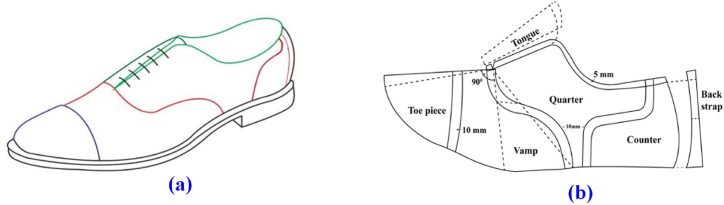
Designed Oxford shoe (a); Oxford Standard construction (b).

The problem stated in this research is primarily how to arrange footwear components in groups regarding material physiognomy without overlap. The upper leathers are a naturally occurring artifact. Their shapes are extremely irregular, and their internal heterogeneity is very common. Moreover, due to aesthetic considerations, grain quality and tensile strength of upper leather, and foot gait drive specific parts of footwear has to be cut from a specific zone viz. vamp is from the butt zone. Consequently, any degree of rotation is restricted by the tightness and stretchiness direction of the upper leather. Hence, the allocated angle of rotations is 0^0^, 45^0^, 90^0^, 180^0^, and 270^0^ for footwear components alignment considering the upper leather physiognomy to obtain maximum utilization of material. Therefore, finding a perfect alignment set with a minimal-area polygonal enclosure is the principal focus of this study to compute upper material consumption with minimum waste. For solving nesting difficulties, at the rudimentary stage, a men's Oxford shoe is designed using the model last size EU 43 (UK 09) as Fig. 1(a) [26,27]**.** The designed shoe was drawn on the masked model last based on the drawing principles of shoe design. For designing, important reference lines and points were manually marked skillfully on the masked last. In the case of locating ballpoints, measurements started from the heel point on the last axis (foot axis) [28]. The Medial Ballpoint (*MB*), Lateral Ballpoint (*LB*), Vamp Point (*VP*), Instep Point (*IP*), Counter Point (*CP*), Back Height (*BH*), Facing Point (*FP*), Vamp Border (*SU*) and Underneath Points of the ankle bone (*UA*) were taken according to equations (1)–(9) [18,28,29].

(1) $$MB=\frac{3}{4}\;SLL$$

(2) $$LB=\frac{13}{20}\;SLL$$

(3) $$VP=\frac{7}{10}\;SLL$$

(4) $$IP=\frac{1}{4}\;SLL$$

(5) $$CP=\frac{1}{5}\;SLL$$

(6) BH=CP+10mm

(7) $$FP=\frac{1}{4}\;SLL+6\;mm$$

(8) $$SU=\frac{1}{4}\;SLL$$

(9) $$UA=\frac{1}{5}\;SLL$$

Where; *SLL* stands for Standard Last Length. The measured points are shown in Table 1. After replicating all these points, the standard construction for the designed shoe was developed according to Fig. 1(b).

**Table 1:** Important points on the last.

Standard last size	*SLL* (mm)	Parameters	Points (mm)
EU 43	286.8	*MB*	215.10
		*LB*	186.42
		*VP*	200.76
		*IP*	71.70
		*CP*	57.36
		*BH*	67.36
		*FP*	77.7
		*SU*	71.7
		*UA*	57.36

**Fig. 2:**
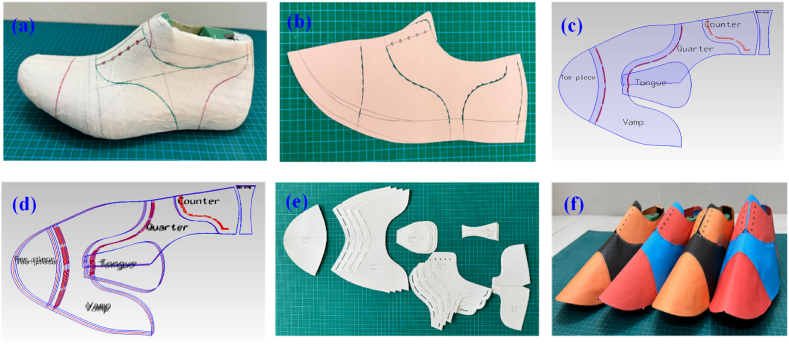
2D Pattern engineering and 3D origami structure for the Oxford shoe.

The cutting patterns were developed following a step-by-step sequential procedure i.e. flattening, mean form, and 2D upper standard construction [18,28]. Subsequently, the manually developed 2D upper standard was transferred to the interface of shoe-master power software through digitization. Later, shell construction, style line smoothening, lasting margin, addition of seam allowances, and piece creation were carried out in the interface of shoe-master software according to pattern engineering [26,29,30]. The upper patterns were graded to reproduce similar shapes of cutting patterns i.e. toe piece, vamp, quarters, tongue, counter, and back strap for the succeeding (EU 41, 42) and preceding (EU 44) sizes of EU 43. The graded patterns were constructed according to the arithmetic grading technique [18,31]. After that, the components are exported to a cutting device known as a plotter to get cutting patterns. Moreover, the origami structures for the shoes were assembled and a footwearist was conserved to justify the dimensional accuracy of patterns. The pattern engineering process is shown in Fig. 2 where; (a) On last drawing; (b) Standard construction, (c) 2D pattern, (d) 2D graded pattern, (e) Complete set of patterns, and (f) Origami structures.

### 2.2 Model formulation

This section presents the step-by-step techniques for developing a mathematical model to calculate footwear upper material consumption. The model formulation started with the tracing of irregular shapes onto standard oversized grid paper; the dimension of 34 × 44 inches per small square unit was 0.01 sq. in. from Geyer Instructional Products, Cincinnati, Ohio, USA. Then, identification of the curved shape of the footwear components along with areas enclosed by the curves on standard grid paper was done. The region confined by a given function *y = f(x)* which is nonnegative and also on the closed interval (*L*_*1*_*, L*_*2*_), then the area of the region bounded by the function *f;* can be determined by $\int_{L_{1}}^{L_{2}}f\left(x\right)\;dx$; where *f(x)* is integrand, *dx* is integrating agent, and the interval (*L*_*1*_, *L*_*2*_*)* is limits of integral function [32]. This fundamental concept of an area bounded by the curve in definite integral calculus was used to calculate the area of the Footwear Components (FCs) to compute the net material per pair of shoes. The integration process was performed in the Excel spreadsheet. Later, nesting optimization with minimum waste was reached through the iteration of multiple orientations for the footwear components to predict gross material consumption. Moreover, actual upper material consumption for the Oxford footwear was also estimated by following the optimum nesting on A-grade corrected grain shoe upper cow leather (Leather S) through the image processing technique. Simple regression analysis and the Pearson correlation coefficient were performed for the proposed model validation.

#### 2.2.1 Data extraction and area calculation

**Fig. 3:**
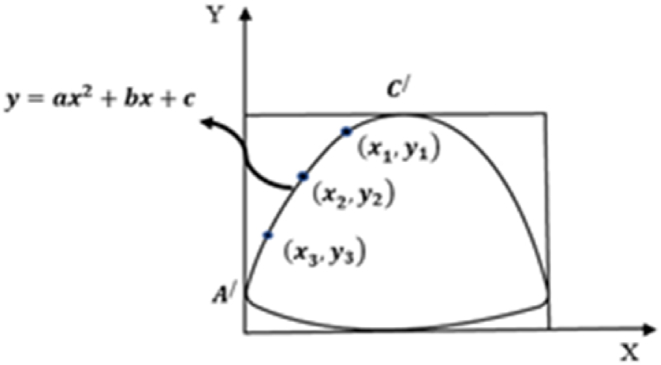
An illustration of a circumscribed toe piece.

In the early phases of data extraction, the developed 2D patterns of the Oxford shoe were traced on grid papers manually considering the first quadrant of the Cartesian coordinate axes. All upper components are then delineated using curves as boundaries and also curves were recognized from sketches for individual pattern area calculation. Shifting of origin was considered for data extraction, area calculation, and limit detection. The prior knowledge of the shoe's last design identified that the shoe fitting lines are quadratic functions viz. *y = f (x) = ax*^*2*^ + *bx* + *c*, where *a ≠ 0; a, b, c ∈ R* and *a, b* are coefficient of *x*^*2*^ and *x* respectively and *c* is constant [33]. All upper parts outlines (unusual shapes) can be defined using the nature of style lines as curves or straight lines. Therefore, the footwear components can be represented as a summation of multiple curve line functions. As the coefficient (a, b) of variables and constant (c) is unknown, hence, for determining the value of coefficient a, b, and constant c; common equations were developed by taking three points on each line through the elimination method. The elimination method is a method used in mathematics to solve a structure of equations. It involves eliminating one variable at a time from the system of equations until only one variable remains [34]. An illustration for the toe piece of footwear was sketched and the curve was represented through equation (10), three points on the curve were taken according to Fig. 3, and the values were placed in equation (10).

(10) $$y=f\left(A^{/}C^{/}\right)=ax^{2}+bx+c$$

(11) $$y_{1}=a{x_{1}}^{2}+bx_{1}+c$$

(12) $$y_{2}=a{x_{2}}^{2}+bx_{2}+c$$

(13) $$y_{3}=a{x_{3}}^{2}+bx_{3}+c$$

The elimination method was applied to equations (11), (12), (13) for the determination of the values of coefficients a, b, and constant c.

(14) $$a=\frac{\left(x_{1}-x_{2}\right)\;\left(y_{1}-y_{3}\right)-\left(x_{1}-x_{3}\right)\;\left(y_{1}-y_{2}\right)}{\left(y_{1}-y_{2}\right)\;\left(y_{1}-y_{3}\right)\;\left(y_{2}-y_{3}\right)\;}$$

(15) $$b=\frac{\left(x_{1}-x_{2}\right)\;\left(y_{1}+y_{3}\right)}{\left(y_{1}-y_{2}\right)\;\left(y_{3}-y_{2}\right)}-\frac{\left(x_{1}-x_{3}\right)\;\left(y_{1}+y_{2}\right)}{\left(y_{1}-y_{3}\right)\;\left(y_{3}-y_{2}\right)}$$

(16) $$c=x_{1}-a{y_{1}}^{2}-by_{1}$$

After that, each pattern was circumscribed securely inside a rectangular polygon. The area bounded by the curve of each segment was calculated by definite integral calculus. Hence, the footwear pattern area was calculated from the difference between the area of the rectangular polygon enclosure and the sum of the area bounded by the curve of each segment. An analogous approach was carried out for other shoe components' area calculations. The following components’ areas were calculated.

##### 2.2.1.1 Toe piece

**Fig. 4:**
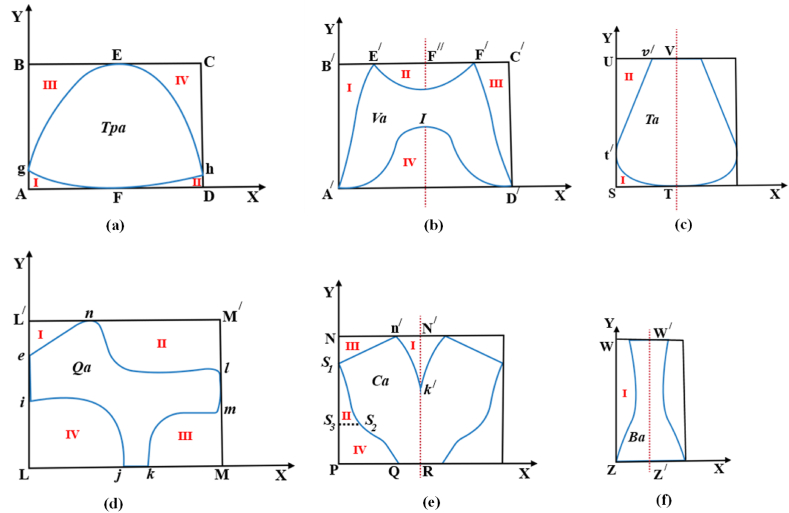
Circumscribed footwear components for area calculation.

The shoe upper component that shields the toe region or the shoe part that covers the metatarsophalangeal joint to the phalangeal area [35]. The toe piece is the most anterior part of the upper material extending forward from the vamp line to the toe of the shoe [36]. The toe piece pattern is asymmetrical due to the length (height) difference of phalanges from the first (big toe) to the fifth phalangeal bones. The position of lateral and medial ballpoints is also different in shoe last due to the irregular foot dorsum [[37], [38], [39]]. The toe piece was traced on grid paper at first and embedded with an ABCD rectangle as depicted in Fig. 4 (a). The area of the toe piece is denoted as *Tpa*. Equations (17), (18) represent the area of the toe piece and the area was calculated from equation (18).

(17) $$T_{pa}=\int_{L_{1}}^{L_{2}}f\left(BC\right)\;dx\;-\;\left[\int_{L_{3}}^{L_{4}}f\left(Fg\right)\;dx+\int_{L_{5}}^{L_{6}}f\left(Fh\right)\;dx+\int_{L_{7}}^{L_{8}}f\left(Eg\right)\;dy+\int_{L_{9}}^{L_{10}}f\left(Eh\right)\;dy\;\right]$$

(18) $$T_{pa}=\int_{L_{1}}^{L_{2}}b\;dx\;-\;\left[\;\int_{L_{3}}^{L_{4}}\left(a_{1}{x_{1}}^{2}+b_{1}x_{1}+c_{1}\right)\;dx+\int_{L_{5}}^{L_{6}}\left(a_{2}{x_{2}}^{2}+b_{2}x_{2}+c_{2}\right)\;dx+\int_{L_{7}}^{L_{8}}\left(a_{3}{y_{3}}^{2}+b_{3}y_{3}+c_{3}\right)\;dy+\int_{L_{9}}^{L_{10}}\left(a_{4}{y_{4}}^{2}+b_{4}y_{4}+c_{4}\right)\;dy\right]$$

Where; *b*, $a_{1}{x_{1}}^{2}+b_{1}x_{1}+c_{1}$**,** $a_{2}{x_{2}}^{2}+b_{2}x_{2}+c_{2}$**,** $a_{3}{y_{3}}^{2}+b_{3}y_{3}+c_{3}$, and $a_{4}{y_{4}}^{2}+b_{4}y_{4}+c_{4}$ stand for the function of *BC* straight line and functions of *Fg, Fh, Eg*, and *Eh* curves respectively*.* Also L_*1*_*, L*_*3*_*, L*_*5*_*, L*_*7*_*, L*_*9*_*,* and *L*_*2*_*, L*_*4*_*, L*_*6*_*, L*_*8*_*, L*_*10*_ represent the lower and upper limits of the integral functions sequentially.

##### 2.2.1.2 Vamp

The part of the shoe upper that covers the instep [35] and also covers the forefoot which spreads to the toe as far as the quarters. The term vamp may also be applied to the material between the toe piece and the quarters [36]. The instep will determine the shape or style of the vamp [40]. The vamp zone on the forepart of the foot is asymmetry in nature due to the irregularity of the foot outline. This unevenness is observed in the dorsal view of the foot anatomical structure which is for the difference in length of phalanges and the different positions of the first and fifth metatarsal-phalangeal joints (ball points of the foot [[37], [38], [39]]. The embedded vamp is illustrated in Fig. 4(b). From the shape of the vamp pattern, it is clear that the E^/^F^/^ concave portion as well as the A^/^ID^/^ convex zone is symmetrical indeed but the whole one is asymmetrical. The area of the vamp is denoted as *V*_*a*_. Equation (19), and (20) denote the area of the vamp, and equation (20) was used to calculate the area of the vamp.

(19) $$V_{a}=\int_{L_{1^{/}}}^{L_{2^{/}}}f\left(B^{/}C^{/}\right)\;dx-\left[\int_{L_{3^{/}}}^{L_{4^{/}}}f\left(A^{/}E^{/}\right)\;dy+2\int_{L_{5^{/}}}^{L_{6^{/}}}f\left(E^{/}F^{//}\right)\;dx+\int_{L_{7^{/}}}^{L_{8^{/}}}f\left(F^{/}D^{/}\right)\;dy+2\int_{L_{9^{/}}}^{L_{10^{/}}}f\left(A^{/}I\right)\;dx\right]$$

(20) $$V_{a}=\int_{L_{1^{/}}}^{L_{2^{/}}}b^{/}\;dx-\left[\int_{L_{3}^{/}}^{L_{4^{/}}}\left(a_{5}y_{5}^{2}+b_{5}y_{5}+c_{5}\right)\;dy+2\int_{L_{5^{/}}}^{L_{6^{/}}}\left(a_{6}x_{6}^{2}+b_{6}x_{6}+c_{6}\right)\;dx+\int_{L_{7}^{/}}^{L_{8^{/}}}\left(a_{7}y_{7}^{2}+b_{7}y_{7}+c_{7}\right)\;dy+2\int_{L_{9^{/}}}^{L_{10}^{/}}\left(a_{8}x_{8}^{2}+b_{8}x_{8}+c_{8}\right)\;dx\right]$$

Where; $b^{/}$, $a_{5}{y_{5}}^{2}+b_{5}y_{5}+c_{5}$**,** $a_{6}{x_{6}}^{2}+b_{6}x_{6}+c_{6}$**,** $a_{7}{y_{7}}^{2}+b_{7}y_{7}+c_{7}$, and $a_{8}{x_{8}}^{2}+b_{8}x_{8}+c_{8}$ stand for the function of B^/^C^/^ straight line and functions of A^/^E^/^, E^/^ F^//^, F^/^D^/^, and A^/^I curves respectively. Also, L_1_^/^, L_3_^/^, L_5_^/^, L_7_^/^, L_9_^/^, and L_2_^/^, L_4_^/^, L_6_^/^, L_8_^/^, L_10_^/^ represent the lower and upper limits of the integral functions.

**Tongue:** A single separate piece that lies where the eyelets of a lace-up shoe are positioned or under lacing to conceal the instep [36]. It is symmetrical along the Y axis [18]. The area of the tongue is denoted as *T*_*a*_. Fig. 4(c) depicts the rectangular enclosure**.** Equations (21), (22) signify the area of the tongue. Equation (22) represents the process of calculating the area of the tongue.

(21) $$T_{a}=2\times \left[\int_{I_{1}}^{I_{2}}f\left(UV\right)\;dx-\left\{\int_{I_{3}}^{I_{4}}f\left(t^{/}T\right)\;dx+\int_{I_{5}}^{I_{6}}f\left(t^{/}v^{/}\right)\;dy\right\}\right]$$

(22) $$T_{a}=2\times \left[\int_{I_{1}}^{I_{2}}b^{//}\;dx-\left\{\int_{I_{3}}^{I_{4}}\left(a_{9}{x_{9}}^{2}+b_{9}x_{9}+c_{9}\right)\;dx+\int_{I_{5}}^{I_{6}}\left(a_{10}{y_{10}}^{2}+b_{10}y_{10}+c_{10}\right)\;dy\right\}\right]$$

Where; *b*^*//*^, $a_{9}{x_{9}}^{2}+b_{9}x_{9}+c_{9}$, and $a_{10}{y_{10}}^{2}+b_{10}y_{10}+c_{10}$ stand for the function of *UV* straight line and functions of *Tt*^*/*^ and *t*^*/*^*v*^*/*^ curves respectively*.* Also, *I*_*1*_*, I*_*3*_*, I*_*5*_*,* and *I*_*2*_*, I*_*4*_*, L*_*6*_*,* represent the lower and upper limits of the integral functions.

##### 2.2.1.3 Quarters

Two sections that form the back, the exterior part of the upper, lay over the instep, and cover the whole midfoot zone to close the shoe as facings. The two quarters are known as the outside quarter and the inside quarter [36,40]. The quarter pattern is the most irregular shape due to the wedge-shaped structure of five squat bones and the principal arch found in the mid-foot region of the anatomical structure of a foot [[37], [38], [39],^41^]. The area of the quarter pattern is denoted as *Q*_*a*_ and Fig. 4(d) depicts its embedded geometry. Equations (23), (24) are the area of the quarter.

Equation (24) was used to calculate the area of the quarter.

(23) $$Q_{a}=\int_{{I_{1}}^{/}}^{{I_{2}}^{/}}f\left(L^{/}M^{/}\right)\;dx-\left[\int_{{I_{3}}^{/}}^{{I_{4}}^{/}}f\left(en\right)\;dy+\int_{{I_{5}}^{/}}^{{I_{6}}^{/}}f\left(nl\right)\;dx+\int_{{I_{7}}^{/}}^{{I_{8}}^{/}}f\left(mk\right)\;dx+\int_{{I_{9}}^{/}}^{{I_{10}}^{/}}f\left(ij\right)\;dx\right]$$

(24) $$Q_{a}=\int_{{I_{1}}^{/}}^{{I_{2}}^{/}}b^{///}\;dx-\;\left[\int_{{I_{3}}^{/}}^{{I_{4}}^{/}}\left(a_{11}{y_{11}}^{2}+b_{11}y_{11}+c_{11}\right)\;dy+\int_{{I_{5}}^{/}}^{{I_{6}}^{/}}\left(a_{12}{x_{12}}^{2}+b_{12}x_{12}+c_{12}\right)\;dx+\int_{{L_{7}}^{/}}^{{L_{8}}^{/}}\left(a_{13}{x_{13}}^{2}+b_{13}x_{13}+c_{13}\right)\;dx+\;\int_{{I_{9}}^{/}}^{{I_{10}}^{/}}\left(a_{14}{x_{14}}^{2}+b_{14}x_{14}+c_{14}\right)\;dx\;\right]$$

Where; *b*^*///*^, $a_{11}{y_{11}}^{2}+b_{11}y_{11}+c_{11}$, $a_{12}{x_{12}}^{2}+b_{12}x_{12}+c_{12}$, $a_{13}{x_{13}}^{2}+b_{13}x_{13}+c_{13}$, and $a_{14}{x_{14}}^{2}+b_{14}x_{14}+c_{14}$ stand for the function of *L*^*/*^*M*^*/*^ straight line and functions of *en, nl, mk*, and *ij* curves respectively*.* Also, *I*_*1*_^*/*^*, I*_*3*_^*/*^*, I*_*5*_^/^*, I*_*7*_^*/*^*, I*_*9*_^*/*^*,* and *I*_*2*_^*/*^*, I*_*4*_^*/*^*, I*_*6*_^*/*^*, I*_*8*_^*/*^*, I*_*10*_^*/*^ represent the lower and upper limits of the integral functions.

##### 2.2.1.4 Counter

The part of the shoe extending around the heel [35]. This part backs the heel around its medial and lateral sides [36,40]. The counter pattern is symmetrical along the Y-axis. The reason for the symmetry lies in the anatomical structure of the rear foot (hind foot) [37,41]. According to the literature, 16 % of the foot length [42] or 40 mm from the heel point [43] along the foot axis on the plantar view of the foot is quite symmetrical. Even from the posterior view of the human foot, it is clear that the two malleolus bones ensure back part symmetry [43]. Therefore, designing a shoe last primarily concentrates on the front part whereas the back part of a shoe last almost remains similar [44]. Fig. 4(e) depicts the embedded geometry of the counter. Equations (25), (26) denote the area of the counter component. The counter component area was calculated from equation (26).

(25) $$C_{a}=2\times \left[\int_{U_{1}}^{U_{2}}f\left(NN^{/}\right)\;dx-\left\{\int_{U_{3}}^{U_{4}}f\left(k^{/}n^{/}\right)\;dy+\int_{U_{5}}^{U_{6}}f\left(S_{1}S_{2}\right)\;dy+\int_{U_{7}}^{U_{8}}f\left(S_{1}n^{/}\right)\;dx+\int_{U_{9}}^{U_{10}}f\left(S_{3}Q\right)\;dx\right\}\right]$$

(26) $$C_{a}=2\times \left[\int_{U_{1}}^{U_{2}}b^{iv}dx-\{\int_{U_{3}}^{U_{4}}\left(a_{15}{y_{15}}^{2}+b_{15}y_{15}+c_{15}\right)\;dy+\int_{U_{5}}^{U_{6}}\left(a_{16}{y_{16}}^{2}+b_{16}y_{16}+c_{16}\right)\;dy+\int_{U_{7}}^{U_{8}}\left(a_{17}{x_{17}}^{2}+b_{17}x_{17}+c_{17}\right)\;dx+\int_{U_{9}}^{U_{10}}\left(a_{18}{x_{18}}^{2}+b_{18}x_{18}+c_{18}\right)\;dx\right]$$

Where *b*^*iv*^, $a_{15}{y_{15}}^{2}+b_{15}y_{15}+c_{15}$**,** $a_{16}{y_{16}}^{2}+b_{16}y_{16}+c_{16}$, $a_{17}{x_{17}}^{2}+b_{17}x_{17}+c_{17}$, and $a_{18}{x_{18}}^{2}+b_{18}x_{18}+c_{18}$ stand for the function of *NN*^*/*^ straight line and functions of *k*^*/*^*n*^*/*^*, S*_*1*_*n*^*/*^*, S*_*1*_*S*_*2*_, and *S*_*3*_*Q* curves respectively*.* Also, *U*_*1*_*, U*_*3*_*, U*_*5*_*, U*_*7*_*, U*_*9*_*,* and *U*_*2*_*, U*_*4*_*, U*_*6*_*, U*_*8*_*, U*_*10*_ represent the lower and upper limits of the integral functions.

##### 2.2.1.5 Back strap

The shoe component that covers the back curve of the quarter's sectional pattern along the back center line. The shape shown here is archetypal to that used in industry, but styles may vary [29]. It is symmetrical along the Y-axis due to the aforementioned reason of counter pattern symmetry. The area of the component is denoted as *B*_*a*_ and its circumscribed geometry is illustrated in Fig. 4(f). Equations (27), (28) represent the area of the back strap. The area was computed from equation (28).

(27) $$B_{a}=2\times \left[\int_{{U_{1}}^{/}}^{{U_{2}}^{/}}f\left(WW^{/}\right)\;dx-\int_{{U_{3}}^{/}}^{{U_{4}}^{/}}f\left(WZ\right)\;dy\right]$$

(28) $$B_{a}=2\times \left[\int_{{U_{1}}^{/}}^{{U_{2}}^{/}}b^{v}dx-\int_{{U_{3}}^{/}}^{{U_{4}}^{/}}f\left(a_{19}{y_{19}}^{2}+b_{19}y_{19}+c_{19}\right)\;dy\right]$$

Where; b^v^, and $a_{19}{y_{19}}^{2}+b_{19}y_{19}+c_{19}$ stand for the function of BC straight line and function of WZ curve respectively. Also, U_1_^/^, U_3_^/^, and U_2_^/^, U_4_^/^ represent the lower and upper limits of the integral functions.

Values of all lower and upper limits and values of variables *x* and *y* were extracted from the standard grid papers where the footwear components were traced and equations (14), (15), and (16) were used to calculate the unknown values of coefficients $a_{1},a_{2},a_{3},...\;a_{19}\text{,}$ $b_{1},b_{2},b_{3},...\;b_{19}\text{,}$ and constants $c_{1},c_{2},c_{3},...\;c_{19}$.

Therefore, net upper material consumption for one pair of footwear is the summation of all upper components' area and it is generally twice of all component's area except quarter parts. Twice of area for the toe piece, vamp, counter, and back strap along with the quadruple area for the quarter part deliver net material consumption. The area of the footwear components *T*_*pa*_*; V*_*a*_*; T*_*a*_*; Q*_*a*_*; C*_*a*_*,* and *B*_*a*_ can be estimated from equations (((18), (20), (22), (24), (26) and (28). Henceforth, net material consumption (*Nc*) for one pair of footwear was calculated from equation (29).

(29) $$N_{C}=2\times \left(T_{pa}+V_{a}+T_{a}+C_{a}+B_{a}\right)+4Q_{a}$$

#### 2.2.2 Generation of nesting variants

The impeccable placement of more than two irregular footwear components onto the material in a logical sequence as compactly as possible but without overlapping is known as nesting. Perfect nesting can minimize interlocking waste and optimize material utilization [26,45]. The pattern placement can be considered for 0^0^, 45^0^, 90^0^, 180^0^, and 270^0^ clockwise rotation with material physiognomy and place similar components from edge to edge to reduce the interlocking gap. Pattern rotation is done based on the center of gravity which was determined through the plumb line method [46].

**Fig. 5:**
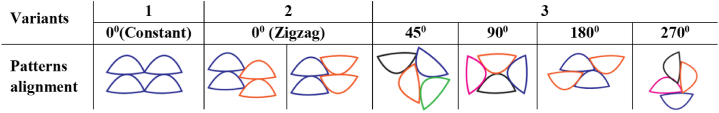
Nesting variants by the orientation of patterns.

The nesting variations were generated by the following nesting rules-one to three associated with the aforementioned rubrics and a typical illustration is shown in Fig. 5.

Variant 1. Zero degree (0^0^) Consistent and components are placed from left to right in horizontal rows with a vertically upward direction.
Variant 2. Zero degree (0^0^) Consistent and components are placed from left to right in a zigzag way with a vertically upward direction.
Variant 3. Components are placed in vertically ascending order from left to right, with the reflection that components from adjacent rows are positioned following the 45^0^ clockwise rotation and other similar patterns are set in the same nesting rubric. This variant 3 is also applied for 90^0^, 180^0^, and 270^0^ clockwise rotation.

However, following the variants; the eight irregular upper components of the designed Oxford footwear size 43 can be experimented. Subsequently, at least eight similar footwear patterns (e.g. four pairs of vamp) were traced on grid paper as compactly as possible without overlapping and embedded by minimal area rectangular enclosure. Later, corresponding utilization ratios can be calculated from equation (30) to find minimum wastes with maximized materials utilization and the best nesting alignment.

(30) $$U_{r}=\frac{\sum_{i=1}^{n}a_{i}}{A_{r}}\times 100$$

Where; *U*_*r*_ indicates the utilization ratio, *a*_*i*_ is the area of the *i*th pattern, *A*_*r*_ is the area of the associated embedding rectangle or the used area of the sheet, and n is the number of the similar *i*th pattern inside the enclosure. Similar footwear components are nested together and need to be cut from alike zones due to color, grain, and nap or other varying features of materials (leather) and also to ensure matching or similar appearance of shoe parts in a pair of shoes [26].

**Fig. 6:**
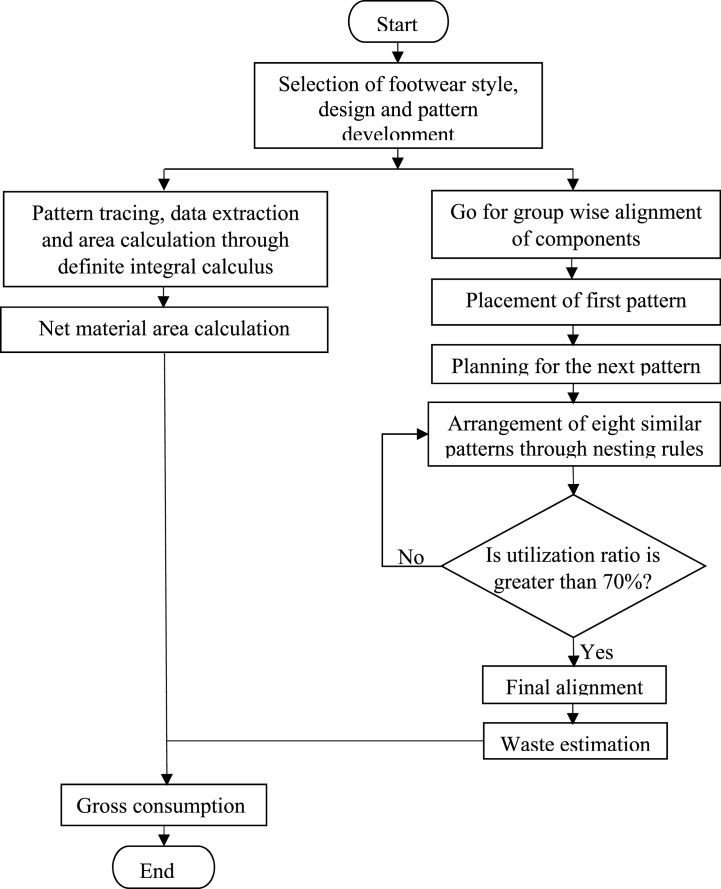
Flow chart of the proposed model.

Many variations of nesting were feasible for each component of footwear, of which the minimal-area rectangular enclosure with maximum utilization ratio has to be cherry-picked. Fig. 6 displays the flow of the proposed model. There are two major parts represented in this flow chart. The first part is footwear design selection, pattern construction, and area computation of each pattern through definite integral to compute net material, and the second is how to place and rotate the irregular shapes and find the final position to search for minimum interlocking waste. At least eight similar component alignments are followed by nesting variations rules introduced in the sub-section 2.2.2 generations of nesting variants in search of 70 % material utilization; otherwise, the algorithm is halted if it cannot find a position to arrange a part. Again, it started with the placement of components to search for a good initial position, the second, third, and minimum eight components. Patterns are gradually moved and rotated without overlapping the shapes for the final sequence of the least embedding rectangle. According to the literature, leather-based footwear manufacturing generates an average of 25–35 % cutting waste [4]. Typically, footwear upper components are cut from large sheets, leaving 30 % or more material to be discarded [8]. Hence, for the expediency of appearance and calculation, the method sets a 70 % utilization margin.

#### 2.2.3 Waste estimation

As the footwear components are the most irregular parts which gives complexity to nesting exertion. Therefore, there must be gaps between interlocked parts that could not be consumed. Consequently, waste is observed in the cutting department of the footwear industry. These wasted materials are commonly referred to as trim or cutting loss. In addition, materials grade and geometry are also responsible for such type of waste [26,47]. So, waste estimation is necessary for calculating material allowance in the material resource planning and procurement department.

**Fig. 7:**
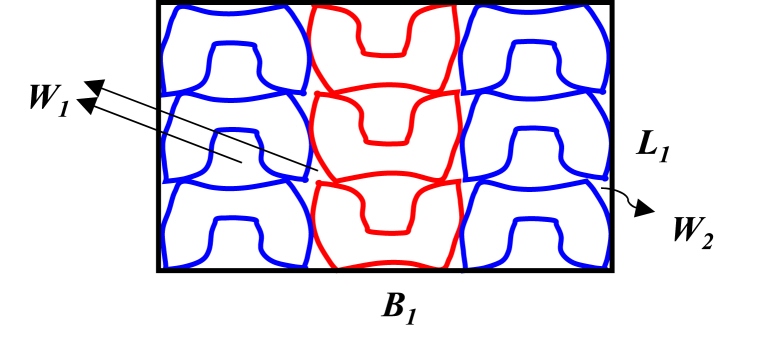
A typical illustration of first and second waste.

Generally, cutting wastes (*W*) is the integration of the first waste (*W*_*1*_), second waste (*W*_*2*_), third waste (*W*_*3*_), and fourth waste (*W*_*4*_). The first waste (*W*_*1*_) i.e. the gap between nested components popularly known as pattern interlocking waste [5,12,26]. The second waste (*W*_*2*_) is known as edge waste (side rest) [12]. An illustration of the first and second wastes is given in Fig. 7, and the third waste (*W*_*3*_) is due to material (leather) imperfection [12,26] or permanent defects on the leather surface that can't be removed through the finishing process.

The best nesting alignment was selected from the percentage of utilization value and from this alignment the first waste was estimated from equation (31) according to the Pick's method of irregular area calculation. The area of an unusual polygon (*A*) can be determined using Pick's theorem by counting the number of square units in its interior (*C*) and on its boundary (*P*) on grid paper, then using the formula; *A = C + P/2 -1* to get the area [48].

The first waste is the summation of all the interlocking gaps for each similarly aligned set of patterns inside the minimal-area enclosure polygon.

(31) $$W_{1}=\left(C_{m}+\frac{p_{m}}{2}-1\right)\;m+\left(C_{q}+\frac{p_{q}}{2}-1\right)\;q$$

Where; *W*_*1*_*, C*_*m*_, *C*_*q*_, *P*_*q*_, *P*_*m*_, *m*, and *q* indicate the first waste, number of complete squares inside the larger interlocking gap of patterns, number of complete squares inside the smaller interlocking gap of patterns, number of squares on the perimeter of the irregular larger, and smaller interlocking gaps, number of total larger interlocking gaps and number of total smaller interlocking gaps of the traced components on grid paper respectively. The second waste was calculated from equation (32).

(32) W2=(L1×B)1−(ns×AS+W1)

Where; *W*_*2*_, *L*_*1*_, and *B*_*1*_ represent the second waste, length, and breadth of an embedded rectangle for the nested components respectively, and *n*_*s*_, *As*, and *W*_*1*_ indicate the number of similar patterns, area of a similar pattern inside the circumscribed polygon, and first waste correspondingly. For the third waste (*W*_*3*_); 3 % of waste for A-grade leather was considered following the five-point rating index of SATRA [26]. Leather is a heterogeneous material. So, wastes for material defectiveness must be considered. Therefore, the third waste was estimated by equation (33).

(33) $$W_{3}=N_{C}\times 3\%$$

**Fig. 8:**
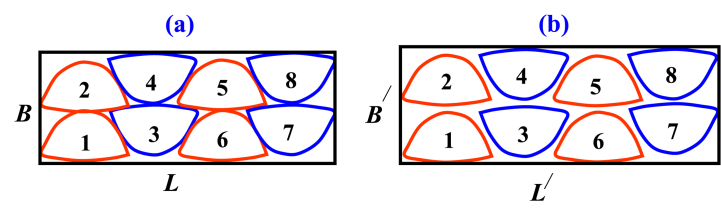
An illustration of drawn geometries for the fourth waste determination.

The fourth waste (*W*_*4*_) is based on the distances between cutting dies [6]. The footwear design and planning department frequently ignored this fourth waste but this must be considered for actual material estimation. For determining the fourth waste; similar four pairs of patterns are traced by keeping a 2.0 mm gap between two adjacent patterns following the alignment which previously gave minimum first waste or maximum material utilization ratio. The fourth waste was determined from the difference in areas of the two embedded rectangles. Equation (34) represents the fourth waste. An emblematic illustration of the fourth waste determination for the toe piece is shown in Fig. 8. A similar procedure was repeated for the other components of footwear.

Compact nesting (a), Nesting with 2 mm gap (b).

(34) W_4_ = (L^/^ × B^/^) – (L × B)

Hence, total waste is the accumulation of all the four types of waste. It was calculated from equation (35).

(35) W = W_1_ + W_2_ + W_3_ + W_4_

### 2.3 Gross consumption

Gross material consumption is how much material along with processing waste necessitated to produce a product [47]. The heterogeneity of leather and cutting rules for specific parts from specific zones lead to unavoidable wastes [12,26,45] but can be reducible. So, gross consumption of shoe upper is the integration of net material consumption along with the wastes generated on nesting of different footwear components onto the material surface for a pair of shoes. It was calculated from equation (36).

(36) G_C_= N_C_ + W

Where; *G*_*C*_*, N*_*C*_, and *W* represent gross material consumption, net material consumption, and total waste respectively. Therefore, equations (29), (35) and (36) were used to compute footwear upper material consumption for a pair of shoes.

## 3 Results and discussion

This research has proposed a mathematical model for the estimation of footwear upper material consumption with rotational flexibility of patterns while placing onto leather following surface physiognomy and compared it to the orthodox method. The research has highlighted statistically significant differences in gross consumption and material utilization ratio compared to the other conventional methods. Nonetheless, the developed model is verified by the nesting of footwear components onto a corrected grain leather (Leather S) and this effort proved to be feasible for the footwear industry by the achievement of good results.

Table 1 depicts the noteworthy points identified on masked last for designing Oxford shoe. These points on the three-dimensional (3D) last are very significant as a pattern is a two-dimensional (2D) depiction of the 3D last which is evident in pattern engineering and dimensional accuracy of footwear components. The *VP, IP*, and the ballpoints (*MB* and *LB*) collectively determine the boundary of the vamp pattern and also ensure the widest part of the foot gets into the shoe properly [37,41] and the *IP* point also indicates the depth of the vamp point which ensure the minimum length of facing and tongue pattern position and opening. The snug fitting of the shoe's rear part ultimately depends on the proper measurements of *BH, CP, and UA* [18]. Therefore, the aforementioned reference points are indispensable parts of footwear pattern engineering to ensure perfection in pattern development.

Table 2 summarizes the individual component's area for the four consecutive sizes (EU 41, 42, 43, 44) of the Oxford footwear and also represents the corresponding net material consumptions obtained through the proposed mathematical model. The size-to-size change of area for individual components was observed clearly in Table 2. It can be comprehended that the observed shifting of the component's area is followed by regular intervals for individual parts from size to size of footwear but is not analogous for all six components of footwear. The area of the vamp is the largest among the components. It is evident from Table 2 that the increment of areas from size to size for the graded components is an arithmetic progression. The average net material consumption per pair of shoes was calculated at 1.58 sq. ft. from Table 2, and the mean value of net material allowance was estimated at 1.36 % for shifting of shoe size.

**Table 2:** Area of footwear components and net consumption.

S.N.	Size	FCs Sym.	ARA sq. ft.	ABC sq. ft.				Area sq. ft.	One pair sq. ft.	*N*_*C*_ sq. ft.
				I	II	III	IV			
1	41	*T*_*pa*_	0.1783	0.0076	0.0079	0.0210	0.0231	0.1188	0.2376	1.5505
		*V*_*a*_	0.3388	0.0273	0.0165	0.0348	0.0201	0.2033	0.4066	
		*T*_*a*_	0.0774	0.0015	0.0053	–	–	0.0638	0.1275	
		*Q*_*a*_	0.2384	0.0062	0.0504	0.0261	0.0408	0.1149	0.4596	
		*C*_*a*_	0.1975	0.0005	0.0079	0.0132	0.0109	0.1326	0.2652	
		*B*_*a*_	0.0399	0.0064	–	–	–	0.0270	0.0541	
2	42	*T*_*pa*_	0.1833	0.0080	0.0077	0.0244	0.0238	0.1195	0.2391	1.5680
		*V*_*a*_	0.3632	0.0335	0.0170	0.0365	0.0271	0.2051	0.4101	
		*T*_*a*_	0.0795	0.0016	0.0061	–	–	0.0642	0.1283	
		*Q*_*a*_	0.2503	0.0075	0.0534	0.0307	0.0421	0.1166	0.4664	
		*C*_*a*_	0.2032	0.0006	0.0086	0.0088	0.0161	0.1349	0.2699	
		*B*_*a*_	0.0400	0.0064	–	–	–	0.0271	0.0542	
3	43	*T*_*pa*_	0.1881	0.0083	0.0080	0.0277	0.0236	0.1205	0.2410	1.5897
		*V*_*a*_	0.3840	0.0274	0.0175	0.0329	0.0403	0.2080	0.4161	
		*T*_*a*_	0.0819	0.0014	0.0069	–	–	0.0652	0.1305	
		*Q*_*a*_	0.2551	0.0087	0.0486	0.0331	0.0462	0.1186	0.4742	
		*C*_*a*_	0.2058	0.0007	0.0080	0.0045	0.0214	0.1368	0.2736	
		*B*_*a*_	0.0400	0.0064	–	–	–	0.0271	0.0543	
4	44	*T*_*pa*_	0.1929	0.0079	0.0093	0.0249	0.0283	0.1225	0.2450	1.6147
		*V*_*a*_	0.4047	0.0328	0.0190	0.0370	0.0427	0.2116	0.4232	
		*T*_*a*_	0.0844	0.0016	0.0074	–	–	0.0664	0.1327	
		*Q*_*a*_	0.2600	0.0077	0.0519	0.0372	0.0423	0.1209	0.4835	
		*C*_*a*_	0.2085	0.0011	0.0114	0.0043	0.0184	0.1379	0.2759	
		*B*_*a*_	0.0400	0.0064	–	–	–	0.0272	0.0544	

*FCs, Sym., ARA, and ABC indicate footwear components, symbols, associated rectangle area, and areas bounded by the curves respectively.

The nesting alignment generated manually by following variant rules for component placement onto leather was sorted out by the pruning technique based on the minimum 70 % material utilization rule of the algorithm already described in the model formulation part. The illustration in Table 3 shows the successful solutions for different components. Analogous results were found for the four consecutive sizes of footwear. The automatic nesting heuristics cannot be implemented blindly due to the heterogeneity of leather and still automatic defect detection is identified as inefficient in the case of leather [49,50].

**Table 3:**
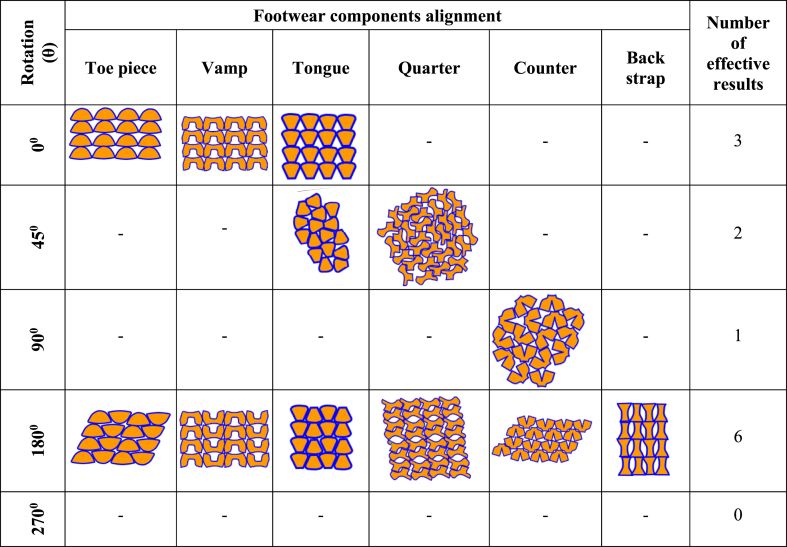
The nesting alignment for Oxford footwear components with minimum waste.

Table 3 represents the successful alignments of patterns on the five specific rotations which are the output of the algorithm set in Fig. 6. It is observed that there are two sets of layouts that satisfy the nest rules except the back strap part. There is no possible solution for the 270^0^ rotation. When eight sets of patterns were being nestled, a material utilization drop of less than 70 % at 270^0^ rotation was noted. This might be because rotation at 270^0^ may provide less than ideal conditions on the material's surface, and even overlap would be feasible. Moreover, it is clear from the illustration in Table 3 that the maximum successful solutions for the nesting of components were from 180^0^ rotation. The general explanation for achieving better results at 180^0^ rotation is that a concave or convex contour by 180^0^ replacement may align the curves sides of the irregular shapes with each other. This arrangement can assist in reducing waste as the curve areas can fit together more closely without leaving spaces. Therefore, this encapsulation of space may reduce space between the edges of shapes which ensures more efficient material utilization.

**Fig. 9:**
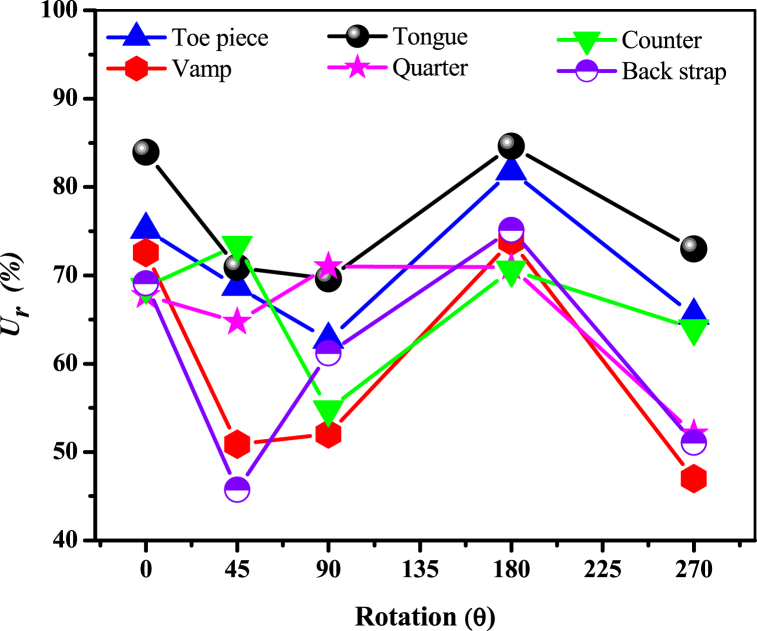
Utilization ratio of the footwear components at different rotations.

The best results among different possible arrangements following the variants rules of the specified rotation were obtained from equation (30) for the four sizes of footwear. Fig. 9 depicts the percentage of utilization for each component's nesting at different orientations for size 43. Then sort out the best from the best pattern nesting alignments. The variation of utilization ratio for different components at different angles is perceived from the utilization graph demonstrated in Fig. 9. The highest (84.62 %) and the lowest (70.62 %) utilization ratios were observed for tongue pattern and counter pattern respectively. This may be the differences in the individual surface areas of footwear components and perhaps the existence of concavity and convexity for a single component. According to Cristina S. et al., 2011 [51], it is due to patterns' configuration and the surface area. From Fig. 9 it is perceived that most of the component's nesting reached the 70 % utilization setup rule at 180^0^ rotation whereas it was different for the quarter pattern (90^0^) and the counter pattern (45^0^).

On the subsequent, from Fig. 9; for the counter pattern, a 45^0^ rotation was the most successful one and that can be due to its single-axis symmetry. This may ensure a balanced as well as coherent rotation that can maintain a logic of direction and harmonization within the irregularity of the leather. Again, as the preferred utilization ratio was also different for quarter pattern nesting; the plausible rationalization can be the quarter component is a highly asymmetrical part among all the FCs. In addition, a 90^0^ rotation can add benefit to material utilization by allowing the quarter pattern's geometry to be nested more efficiently and closely to the material's dimensions. Furthermore, it can be said that highly asymmetrical shapes of quarters may fit better inside the boundaries of the unusual material sheet (leather) and can reduce the quantity of unused material and minimize waste. Again, the drastic reduction of material utilization at 270^0^ rotation for each pattern has been noticed in Fig. 9. The possible cause for this is that rotation at 270^0^ could lead to suboptimal within the material surface and lead to the intersection of components outlines. Therefore, from the results represented in Fig. 9; the best nesting alignment of the individual footwear component was picked up for corresponding wastes estimation. The first, second, and fourth wastes for individual components were the output of equations (31), (32), (33) respectively, and total waste was calculated from equation (34) for the four sizes of footwear.

**Fig. 10:**
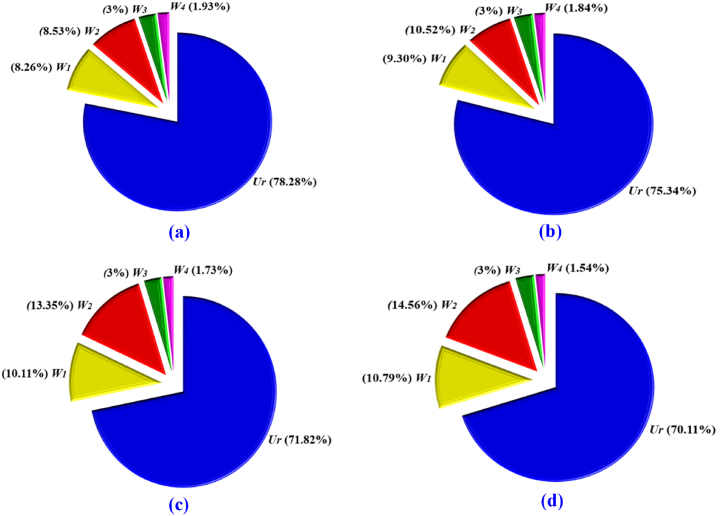
Proportion of material utilization and wastes through the proposed model.

Table 4 represents the four types of estimated waste along with the total waste per pair of shoes for the four consecutive sizes of the Oxford footwear. Results presented in Table 4 showed that with the increase in footwear size number, the optimum wastes for the size-to-size progression of footwear gradually increased except for the fourth waste (*W*_*4*_). The cause for the first (*W*_*1*_) and second (*W*_*2*_) waste can be addressed as the geometry of the unusual components’ shape which plays a vital role. In addition, if the material also possesses the shape of irregular contours, protrusions, or spaces, increasing its space may lead to inefficiencies in nesting, as it may create gaps or overlaps with neighboring shapes. Nevertheless, the third waste is 3.0 % of net material and the fourth (*W*_*4*_) waste was constant for each shoe size. The reasonable illumination in favor of the constant fourth waste value is that all the components are always nested side by side by keeping a 2 mm constant gap from edge to edge. The percentage values of each kind of waste along with utilization for all four sizes of shoes are illustrated through pictographs in Fig. 10; where (a), (b), (c), and (d) designate the data for the footwear size EU 41, 42, 43, and 44 respectively. The first and second wastes propagate as the areas of the components increase which is influenced by the shape of the FCs. In order to reduce first and second wastes; it is suggested to simplify the shape of components and a larger surface area of leather is recommended to consider. The percentage of the third waste is constant as the similar grade (A grade) of material is considered. The fourth waste decreases and this may be due to the growth of surface areas of the footwear components. The utilization ratio of the material varies from 70.11 % to 78.28 %; the disparity of the utilization ratio for the four sizes is clearly illustrated in Fig. 10. Therefore, the gross consumption (*G*_*C*_*)* is shown in Table 5 for the four consecutive sizes of footwear which was calculated from equation (34) by utilizing the value of *N*_*C*_ and *W* from Table 2, Table 4. These values were the predicted consumption for the designed Oxford footwear through this proposed model.

**Table 4:** Waste estimation for a single pair of shoe.

Size	Wastes sq. ft.				Total wastes/pair sq. ft.
	W_1_	W_2_	W_3_	W_4_	W
41	0.1636	0.1689	0.0465	0.0384	0.4174
42	0.1936	0.2189	0.0471	0.0384	0.4980
43	0.2236	0.2955	0.0477	0.0384	0.6052
44	0.2525	0.3641	0.0484	0.0384	0.7034

**Table 5:** Comparison of actual and predicted upper material consumption.

Size	Actual consumption/pair sq. ft.	Predicted Consumption/pair sq. ft.	Absolute Error %
41	2.1279	1.9679	7.5191
42	2.2410	2.0660	7.8090
43	2.4099	2.1949	8.9215
44	2.7031	2.3181	14.242

### 3.1 Model validation

**Fig. 11:**
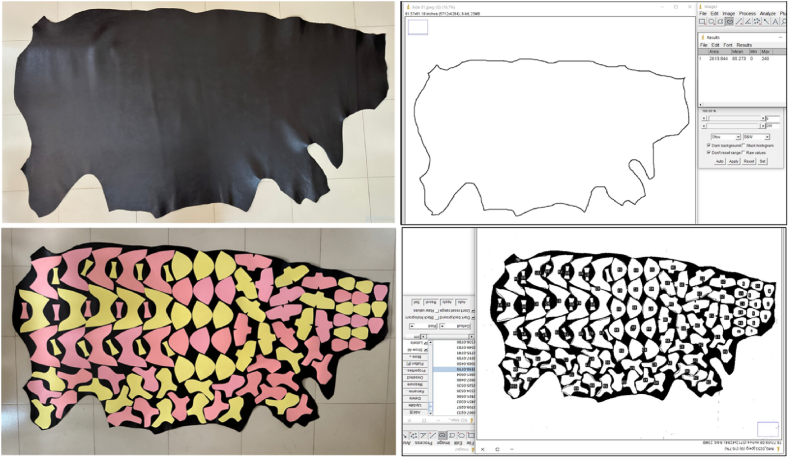
Actual consumption computation through ImageJ software.

Validation is an indispensable phase for any computational model to determine how sound the implemented archetypal resembles reality [52]. For the substantiation of the proposed model, “A” grade corrected grain shoe upper (Leather S) was collected from a renowned export-oriented footwear industry situated at Savar, Dhaka, Bangladesh. The sample origin is cowhide. The thickness and area of the sample were 1.6 mm and 18.04 sq. ft. respectively. The cutting value of the sample was achieved a 17.46 sq. ft. For measuring sample thickness SATRA STD 483 (ISO 2589) thickness gauge meter was used and the area was measured through Image J software by capturing an image of the sample with a DSLR EOS80D digital camera with 1X magnification. This camera was also used with a constant illumination system for capturing all other images. After that, more than seven pairs of the Oxford shoe's FCs (105 patterns) for size 43 were placed according to the priority zones (i.e. vamp and toe piece from butt area on leather) onto the leather S by following the nesting arrangement for individual components of the proposed model that was provided in Fig. 11. Consequently*,* the shoe nesting alignment along with a calibrated scale onto the leather S was captured at jpg format with 4284 x 5712 pixels. The actual consumption calculation was done by the image processing technique using the ImageJ software. The sequential steps are followed as original image acquisition; scale calibration, transformed image into 8-bit greyscale then thresholding was applied; selected the region of interest (ROI), and finally measured the areas**.** An analogous approach was also carried out for 41, 42, and 44 sizes onto the same leather S. Here, only the size variation is considered while maintaining the original design of the Oxford shoe developed in section 2.1. Constant design is used to prevent influencing of other parameters in the consumption calculation. The four consecutive sizes with the same design were selected to investigate the nature of material shifting and the scenario of waste fluctuations. It is noted that there is a good arithmetic progression exists like size-to-size changes of net material consumption provided in Table 2. Additionally, this demonstrates that pattern grading is accurate. An illustration of the actual consumption determination through ImageJ software onto the leather S is shown in Fig. 11. The actual and anticipated footwear upper material consumption along with the percentage of absolute error is demonstrated in Table 5.

The size-to-size material allowance is 3.98 % on average achieved through this model**.** Actual and estimated consumption differed because the model only took into account the minimal-area polygonal enclosure surface profile but leather surfaces are usually the most unusual ever spotted. However, in this study, the estimated consumption for the 42 size Oxford shoe (2.066 sq. ft.) was achieved by the proposed model that fulfills the requirements of Italian standards [12]. The average upper material consumption per pair of shoes was predicted at 2.14 sq. ft. from Table 5.

**Fig. 12:**
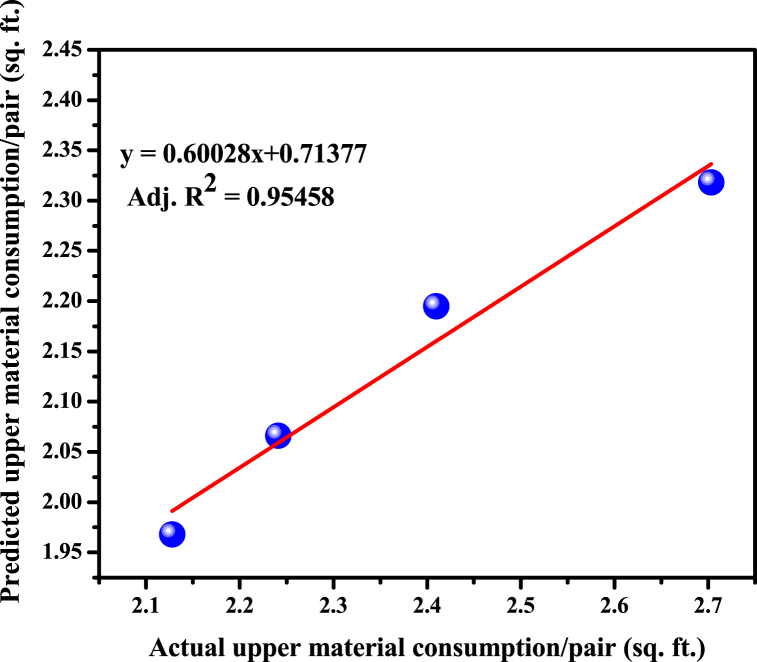
A regression line plot between predicted and actual material consumption for shoes.

The actual and predicted values of footwear upper leather per pair for the aforementioned four sizes of Oxford shoe were compared by fitted line plot which is given in Fig. 12.

The calculated mean absolute error was 9.62 %. The adjusted R-square value and R-square value of the fitted line are 0.95458 and 0.9697 respectively which designate that the variation between the two data sets is in a state of good agreement. Here, the adjusted R-square value is considered due to the small sample size and to avoid overfitting issues. The high value of the adjusted R-square validates the accuracy of the developed model and elucidates that the proposed model has accounted for all the variables on which the upper material requirement hinges. In addition, the very small Residual Sum of Squares (RSS) value is 0.00211 indicates that the model does an outstanding job of illuminating the variance in the data. Furthermore, Pearson's correlation between the actual and predicted values of upper material consumption is 0.98474 with a P-value of 0.0152 (p < 0.05) indicating a very strong ability and correctness of the model.

### 3.2 Comparative study

As far as the author's knowledge, there is a dearth of pertinent work with a complete data set for an Oxford shoe from individual component's area to corresponding wastes for each component especially along with the fourth waste. Moreover, footwear upper design with construction, size, and material grades are also prime factors in the comparison study. Subsequently, a direct comparison with other concepts or methods of consumption becomes challenging. However, for comparison, the experimental work by H. Florentina and M. Aura [53] based on the RSM concept using AutoCAD 2006 software is considered, although the particular shoe size and material's grade are not mentioned in this article. The average percentage of total waste for the aforementioned software-based model is achieved at 26.72 %; whereas it is 24.66 % for the proposed model. Additionally, upper material consumption for 42 size of an Oxford shoe is considered for comparison. Here, the reason behind the selection of 42 size is due to some data availability in the literature [12,18]. Henceforth, a comparison is presented between Thornton's estimation, UNIDO report-2003, and the proposed mathematical model in Table 6; whereas both the system is based on the RSM method. The RSM method considers scale (*S*) area rather than individual pattern area where scale area is the summation of pattern area and corresponding first waste.

**Table 6:** Comparison of the proposed mathematical model with other models.

Parameters	Florentina [53]	Thorton [18]	UNIDO [12]	Proposed model	
**Shoe type**	–	Oxford	Oxford	Oxford	
**Size**	–	42	42	42	
**Components/pair**	14	12	–	14	
***N***_***C***_**(sq. ft./pair)**	–	–	–	1.568	
***G***_***C***_**(sq. ft./pair)**	–	2.062	2.314	2.066	
***U***_***r***_**(%)**	73.28	–	–	75.34	
**S/pair**	–	1.60	–	1.76	
**Waste** (**%)**	***W***_***1***_	21.48	–	–	9.30
	***W***_***2***_	–	17.65		10.52
	***W***_***3***_	–	3	–	3
	***W***_***4***_	–	–	–	1.84
**Nesting orientation**	Two (0^0^, 180^0^)	Two (0^0^, 180^0^)	Two (0^0^, 180^0^)	Five (0^0^, 45^0^, 90^0^, 180^0^, 270^0^)	

Additionally, the first waste is not determined separately in the RSM method [23]. This proposed model introduces the first waste determination technique. So. Scale area can also be calculated through this suggested approach. The comparatively higher value of “*S”* in the case of the suggested model is due to the consideration of the first and second waste inside the embedded enclosure of the nesting components.

This proposed model saves 2.06 % more materials than that of Florentina [53]. The comparative study reveals that the fourth waste is frequently ignored in the mentioned three methods [12,23,53]. The proposed model considered a minimum of eight similar patterns in the nesting process along with five angles of orientation to minimize interlocking waste whereas the other two works consider a smaller number of patterns and only two types of alignment for pattern nesting. In this proposed model, the actual scenario of the nesting onto leather was observed through the image processing technique and also validated by statistical analysis techniques known as linear regression analysis and Pearson correlation coefficient determination.

## 4 Conclusion

In this analysis, a new approach has been conducted to calculate upper material consumption for a complete set of upper components from a designed Oxford shoe with four consecutive sizes considering nesting challenges. The model utilizes the concept of minimal-area polygonal enclosure along with multiple orientations of patterns onto material and irregularity of component contour. The accuracy level of the proposed model for the prediction of shoe upper material consumption was 95.46 %. It is established that this developed model can be applied to compute upper material consumption precisely. The required upper leather per pair of footwear (Men's Oxford style) varies between 2.0 and 2.4 sq. ft. The estimated values meet the standard set by UNIDO for both the Indian sub-continent and Italy. The results of the proposed approach revealed that the upper material consumption depends on shoe size, material grade, components, and material profile. The average material utilization achieved through this model was 73.89 % which is outstanding in comparison to similar data found in the literature. The proposed model reduces the average material requirements of shoes by 2.06 %. Consequently**,** this high usability and real-world experiments with more than 105 numbers of patterns (7.5 pairs) onto leather allow the applicability of the model in the footwear manufacturing industry. Besides, the results of actual consumption by image processing technique through ImageJ software signifies a support for the robustness of the developed model's parameterization for usability in the footwear industry to compute material consumption easily and perfectly.

Apart from the advantageous results, this study has certain limitations that could be addressed in future research. First of all, the model considered a minimal-area polygonal enclosure algorithm for computation purposes in the case of mostly irregular leather. Secondly, as normally conferred in the literature, diverse designs of similar footwear with the same size and grades of upper leather are two major parameters that are highly likely to result in different solutions. So, in future research, other designs of footwear and other grades of leather will be investigated. Finally, the nesting heuristics abridged to this analysis are outlying from exhaustive; an extensive diversity of in-house heuristics based on this concept with integration of others heuristics can progress towards further solutions.

## Funding statement

This research did not receive any specific grant from funding agencies that operate in the public, commercial, or not-for-profit sectors.

## Data availability statement

The authors do not have permission to share data.

## Additional information

No additional information is available for this paper.

## CRediT authorship contribution statement

**Muhammad Naimul Hasan:** Writing – original draft, Methodology, Investigation, Formal analysis, Data curation. **Md Elias Uddin:** Writing – review & editing, Supervision, Conceptualization. **Anower Jahan Tamanna:** Writing – review & editing, Investigation, Formal analysis, Data curation.

## Declaration of competing interest

The authors declare the following financial interests/personal relationships which may be considered as potential competing interests: Dr. Md. Elias Uddin reports was provided by Khulna University of Engineering and Technology. If there are other authors, they declare that they have no known competing financial interests or personal relationships that could have appeared to influence the work reported in this paper.
